# Transient B cell lymphopenia revealed by KRECs newborn screening: Post‐screening referral strategies, clinical course, and follow‐up

**DOI:** 10.1111/pai.70421

**Published:** 2026-07-07

**Authors:** Guarnieri Valentina, Drago Enrico, Pelosi Caterina, Boscia Silvia, Schena Francesca, Traverso Monica, Aloi Concetta, Salina Alessandro, Palmeri Serena, Cassanello Michela, Maghnie Mohamad, Astorino Valeria, Lodi Lorenzo, Azzari Chiara, Volpi Stefano, Ricci Silvia

**Affiliations:** ^1^ Pediatric Immunology Unit Meyer Children's Hospital IRCCS Florence Italy; ^2^ Department of Neuroscience, Rehabilitation, Ophthalmology, Genetics Maternal and Child Health (DINOGMI), University of Genoa Genoa Italy; ^3^ Clinical and Experimental Immunology IRCCS Istituto Giannina Gaslini Genoa Italy; ^4^ Gene Therapy Program, Dana Farber/Boston Children's Cancer and Blood Disorders Center Harvard Medical School Boston Massachusetts USA; ^5^ Department of Health Sciences University of Florence Florence Italy; ^6^ Rheumatology and Autoinflammatory Diseases Unit IRCCS Istituto Giannina Gaslini Genoa Italy; ^7^ UOC Medical Genetic IRCCS Istituto Giannina Gaslini Genoa Italy; ^8^ LABSIEM (Laboratory for the Study of Inborn Errors of Metabolism), Pediatric Clinic and Endocrinology Unit IRCCS Istituto Giannina Gaslini Genoa Italy; ^9^ Biochemistry, Pharmacology and Newborn Screening Unit, Central Laboratory of Analysis IRCCS Istituto Giannina Gaslini Genoa Italy; ^10^ Pediatric Clinic and Endocrinology Unit IRCCS Istituto Giannina Gaslini Genoa Italy

**Keywords:** B‐cell, children, IEIs, KREC, late‐maturation, NBS

## Abstract

**Background:**

Newborn screening (NBS) for inborn errors of immunity increasingly uses T‐cell receptor excision circles (TREC) and, in some programs, Kappa‐deleting recombination excision circles (KREC) to detect early T‐ and B‐cell lymphopenia. While TREC‐based screening is well established, the significance and management of isolated low KREC remain unclear.

**Objective:**

To evaluate the implications of two different regional post‐screening algorithms for isolated low KREC and to characterize the clinical course, immunological profile, and follow‐up of term newborns with transient B‐cell lymphopenia.

**Methods:**

We performed a retrospective multicenter study of term newborns with isolated low KREC identified through NBS, confirmed B‐cell lymphopenia, and subsequent normalization during follow‐up. KREC levels, B‐cell counts, and serum immunoglobulins were assessed longitudinally by RT‐PCR and flow cytometry.

**Results:**

Eighteen newborns were enrolled. At the first evaluation (V1; mean age 13.5 days), all had marked peripheral B‐cell lymphopenia (CD19^+^ ≤ 2%; mean 54 cells/μL), although repeat dried blood spot (DBS) testing already showed KREC values above the diagnostic cutoff in 78%. By the second visit (V2; mean age 50 days), B‐cell percentages and absolute counts normalized in all infants, with emerging IgA and IgM production, and normal KREC on whole blood. No infectious or immunological complications were recorded over 39.5 person‐years of follow‐up (mean 2.3 ± 1.7 years).

**Conclusion:**

Isolated low KREC at birth may identify newborns with transient B‐cell lymphopenia that resolves during early infancy. Repeat KREC testing on a second DBS before referral may represent a pragmatic triage step to reduce unnecessary immunological evaluations, while preserving early assessment for newborns with persistent abnormalities. Prospective studies are needed to refine post‐screening strategies.

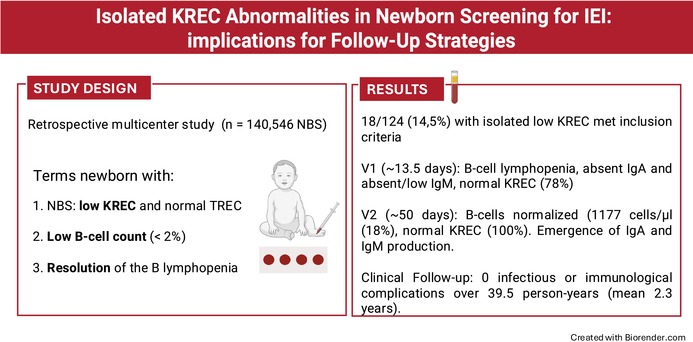

**Abbreviations**
ADA: Adenosine deaminase
BTK: Bruton tyrosine kinase
CT: Cycle Threshold
DBS: Dried blood spots
IEI: Inborn errors of immunity
KREC: Kappa‐deleting recombination excision circles
NBS: Newborn screening
PNP: Purine‐nucleoside phosphorylase
REDCap: Research Electronic Data Capture
RT‐PCR: Real Time polymerase chain reaction
SCID: Severe Combined Immunodeficiency
TMS: Tandem Mass Spectrometry
TREC: T‐cell receptor excision circles
V1: Referral visit 1
V2: Visit 2

**Key message**

TREC‐based SCID screening is established, but the clinical significance and optimal follow‐up of term newborns with isolated low KREC remain unclear. This multicenter cohort characterizes term newborns with isolated low KREC and B‐cell lymphopenia, that during the follow up resolved, defining the time course and, detailing their clinical and immunologic features, documenting follow‐up through telemedicine. Findings suggest that early KREC testing on a second DBS before referral may help reduce unnecessary immunological evaluations and support the value of integrating clinical evaluation during early follow‐up, while underscoring the need for larger prospective studies to inform follow‐up strategies.

## 1 INTRODUCTION

Inborn errors of immunity (IEIs) encompass a varied spectrum of inherited disorders that induce immune dysregulation and deficiency. They lead to an increased vulnerability to life‐threatening infectious diseases, severe autoimmune and malignant conditions, as well as allergies, often resulting in elevated mortality during the early years of life.^1^, ^2^ The International Union of Immunological Societies Expert Committee's 2024 Classification Update details 555 monogenic defects associated with IEIs.^1^ The proposition for newborn screening (NBS) for Severe Combined Immunodeficiency (SCID) emerged in the early 2000s, driven by data indicating a higher survival rate for children undergoing hematopoietic cell transplantation when performed at less than 3.5 months of age and in the absence of pre‐transplantation infections.^3^

Tuscany had pioneered the introduction of NBS for IEIs in Italy, while Liguria subsequently launched a pilot program in 2021. This screening method employs Tandem Mass Spectrometry (TMS) to detect both early and late‐onset forms of adenosine deaminase (ADA) and purine‐nucleoside phosphorylase (PNP) SCID. Moreover, multiplex real‐time polymerase chain reaction (PCR) is utilized to quantify T‐cell receptor excision circles (TREC) and kappa‐deleting recombination excision circles (KREC) from dried blood spots (DBS) to detect respectively thymic and bone‐marrow output.^4^, ^5^ TREC are circular double‐stranded DNA fragments generated during the rearrangement of T‐cell receptor gene and they serve as molecular markers indicative of normal T lymphopoiesis. Similarly, KREC are DNA produced during B‐cell receptor rearrangement, reflecting normal B lymphopoiesis.

While TREC‐based screening for SCID is well established,^6^, ^7^, ^8^ the role of KREC for identifying humoral or combined defects remains a matter of ongoing discussion, given variability in algorithms and false‐positive rates. Implementation choices must balance specificity, timeliness of referral, family burden, and cost‐effectiveness within each health system.^9^, ^10^, ^11^, ^12^, ^13^

In parallel, data are expanding on non‐IEI conditions associated with low TREC (e.g., prematurity, placental insufficiency, critical illness, transient idiopathic T lymphopenia),^14^, ^15^, ^16^, ^17^, ^18^, ^19^, ^20^ whereas the evidence base for isolated low KREC and its clinical implications is comparatively limited beyond specific contexts such as maternal immunosuppression during pregnancy.^9^, ^10^, ^12^

This gap underscores the need to report non‐IEI related cases of abnormal KREC and to improve the definition of standardized post‐screening pathways for newborns flagged by isolated abnormal KREC on DBS, including indications and timing for clinical assessment, peripheral blood immunophenotyping and follow‐up, as well as recommendations for vaccination counseling and early infection‐prevention advice. In the present report, we describe cases of transient B cell lymphopenia in newborns recalled at KREC screening and discuss implications for newborn screening programs.

## 2 METHODS

### 2.1 Study design

This retrospective, multicenter study analyzes NBS data for IEIs from the Tuscany and Liguria regions covering the periods January 2019 to May 2024.

Samples were collected on Guthrie Cards through a heel prick test, performed within 48–72 h from birth. All specimens were analyzed by Real Time Polymerase Chain Reaction (RT‐PCR) for TREC and KREC. Data were collected and managed using REDCap (Research Electronic Data Capture) and analyzed retrospectively.

### 2.2 TREC and KREC assay

#### 2.2.1 DNA extraction

*Tuscany protocol*: 3.2 mm punches were incubated with 25 μL lysis reagent (DNA Extract All Reagent Kit, REF 4402599, Applied Biosystems) for 10 min at 50°C. Lysis was stopped with 25 μL stabilizing reagent, followed by centrifugation at 2000 rpm for 2 min.

*Liguria protocol*: DNA extraction and RT‐PCR were performed using the Eonis™ SCID‐SMA kit (Revvity, Wallac Oy, Turku, Finland) following the manufacturer's standard protocol.

#### 2.2.2 RT‐PCR analysis

*Tuscany protocol*: RT‐PCR for TREC and KREC was performed in a final volume of 15 μL (TaqMan Fast Advanced Master Mix, 0.5 μM primers and probes, 5 μL DNA). Cycling: 95°C for 20 s, followed by 45 cycles of 95°C for 1 s and 60°C for 20 s. Four controls were included in each run. Diagnostic KREC cutoff: 231 copies/10^5^ cells.

*Liguria protocol*: Quantification of KREC was performed by Multiplex real‐time PCR using an Eonis SCID‐SMA kit (Revvity, Wallac Oy, Turku, Finland). The test quantified TREC and KREC concentration and detected SMN1 exon 7 (and exons 7–8) deletions for SMA screening. *RPP30* served as the endogenous housekeeping gene. Population‐based Ct cutoffs were derived from the 1st percentile of 3000 healthy newborns. KREC cutoff: 108 copies/10^5^ cells. Internal QC monitored mean Ct values; external QC was performed via the CDC Newborn Screening Quality Assurance Program (CDC‐NSQAP, Atlanta, GA, USA).

### 2.3 Diagnostic procedure

#### 2.3.1 Post‐screening algorithms for isolated abnormal KREC

*Tuscany protocol*: Samples characterized by a KREC copies number below the established cutoffs underwent a retest on the same DBS. If the KREC value remained below the cutoff, a referral visit (V1) for clinical assessment and hematochemical evaluation was scheduled within 2–3 weeks of age.

*Liguria protocol*: Samples characterized by a KREC copies number below the established cutoffs underwent retesting on the original DBS within 24–48 h. If confirmed, a second DBS was collected at the birth center within 2–3 weeks of age. Persistent KREC values below the cutoff on the second DBS prompted referral to the immunology center for V1 evaluation.

#### 2.3.2 Laboratory test performed during referral visit

KREC were evaluated during the V1 on a new DBS (DBS_V1) through the same procedure and cutoff described above. At V2 and any subsequent visits, KREC measurements were performed on whole blood. Flow cytometry with a standard TBNK panel, including CD45, CD3, CD4, CD8, HLA‐DR, CD19, CD16, and CD56, was used to analyze the main lymphocyte populations. B‐cell differentiation was assessed by using CD45 and CD19. In case of absent KREC, Bruton's tyrosine kinase (BTK) expression was assessed when requested. Samples acquisition was performed using FACS Lyric (BD Biosciences) flow cytometer. Data were analyzed with FACS suite (BD Biosciences) software. After evaluation of the immunophenotype results, further investigations can be conducted as second‐level test.

#### 2.3.3 Genetic analysis

If screening result was confirmed by flow cytometry or syndromic features were present at V1, genetic testing was performed after informed consent using a trio‐based extended exome panel for combined immunodeficiency/SCID and primary antibody deficiencies related genes (WES_v2, Sophia Genetics) on an Illumina NextSeq platform.

#### 2.3.4 Follow up

In newborns with confirmed low KREC values at the V1 and concomitant B‐cell lymphopenia detected by flow cytometry, an in‐person follow‐up visit was scheduled for 4–5 weeks later. The timing of subsequent visits was tailored according to B‐cell lymphopenia time of resolution. After normalization of B‐cell counts, all patients were monitored via telemedicine to maximize the overall duration of follow‐up (expressed in patient‐years) as part of routine clinical practice. During telephone‐based follow‐up, families were asked a set of open‐ended, qualitative questions aimed at capturing relevant changes in the child's clinical status since the previous assessment. These included questions addressing: (i) overall growth and developmental progress compared with age expectations; (ii) occurrence of infections, with particular attention to frequency, severity, and need for antibiotic treatment; (iii) any episodes suggestive of severe or invasive infections; (iv) hospital admissions or emergency department visits related to infectious complications; and (v) any new or ongoing clinical concerns raised by caregivers.

### 2.4 Inclusion and exclusion criteria

Inclusion criteria were the following: (1) term newborns with isolated KREC values below the center‐specific cutoff at birth (see methods section) AND (2) newborns presenting with low B‐cell count (<2%) detected through flow cytometry in whole blood during their V1 AND (3) resolution of the B lymphopenia on flow cytometry observed during the subsequent immunological follow‐up.

The exclusion criteria included: (1) prematurity, low birth weight, and maternal pregnancy immunosuppression therapy, which require special NBS protocols; (2) newborns referred for both TREC and KREC abnormality at birth and/or ADA/PNP metabolites; (3) true forms of agammaglobulinemia that persist over time, regardless of whether a genetic diagnosis was identified.

Four newborns with genetically confirmed agammaglobulinemia (true positive cases) were instead analyzed separately as disease controls.

### 2.5 Case analysis and parameter assessment

Examination of selected cases encompassed a thorough analysis of diverse parameters, comprising gender, ethnicity, gestational age, birth weight, detailed pregnancy information, and family medical history. Laboratory data, including KREC counts on Dried Blood Spots (copies/10^5^cells), serum immunoglobulin levels (IgG, IgA, IgM), and B lymphocyte subset assessments on whole blood, were analyzed at different time points for all cases:

- *Time 0*: abnormal KREC levels (<231 copies/10^5^cells for Tuscany and <108 copies/10^5^cells for Liguria) on DBS at birth along with normal TREC value set the baseline for subsequent analyses.
- *Referral visit (V1)*: evaluation of KREC levels on new DBS (copies/10^5^cells), immunoglobulins levels and B/T lymphocyte subsets in whole blood.
- *Visit 2 (V2) and subsequent visit if available*: evaluation of KREC levels measured on whole blood, immunoglobulin levels, and B/T lymphocyte subsets.
- *Telemedicine follows up*: families were contacted to inquire about any new clinical events since the last visit and to provide remote monitoring

Clinical features potentially indicative of immunologically relevant conditions were identified during V1 and/or during subsequent visits.

The study was approved by the pediatric Ethics Committee of Meyer Children's Hospital (ID number: 111/2023 “NBSxIEIs study”) and by Liguria Regional Ethics Committee (Prot. DBSSCREE20 CER Liguria: 366/2020; DB id 10,679; December 16, 2020). Written, informed consent from the patients' parents or legal guardians was acquired before data collection when possible, and other data were anonymized.

### 2.6 Statistical analysis

Statistical analyses were performed using SPSS Software. A comprehensive descriptive statistical analysis was executed to summarize the variables of interest, providing insights into the distribution of the data.

## 3 RESULTS

### 3.1 Characteristics of selected cases

**FIGURE 1:**
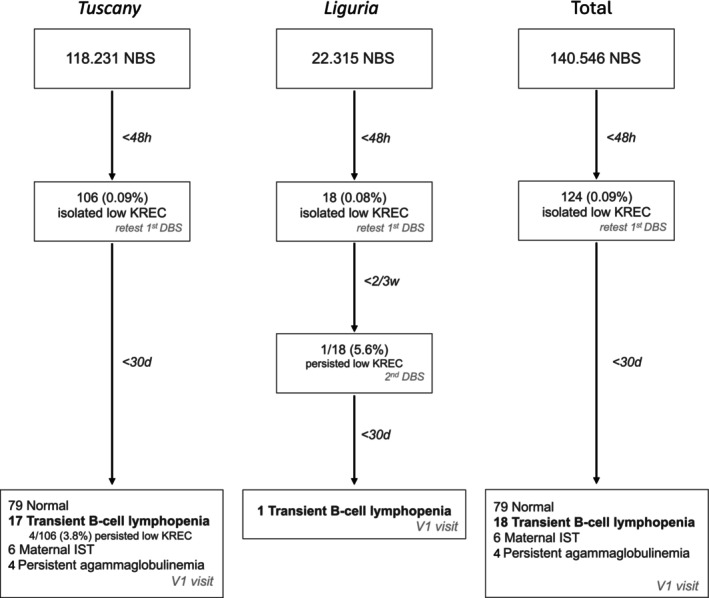
Flow diagram of newborn screening outcomes for isolated low KREC in the Tuscany and Liguria cohorts. DBS, dried blood spot; IST, immunosuppressive therapy; KREC, kappa‐deleting recombination excision circles; NBS, newborn screening; V1, referral visit at the tertiary immunology center.

The screening pathway and recall outcomes of the two regional cohorts are summarized in the flow diagram shown in Figure 1. A total of 140.546 newborns underwent NBS for IEI in Tuscany and Liguria from January 2019 to May 2024. Among these, 124 newborns were recalled for isolated low KREC. Of these, 18/124 (14,5%) were term newborns with confirmed B‐cell lymphopenia at V1 and subsequent normalization during follow‐up, and were therefore included in the study. The characteristics of these selected cases, including sex, ethnicity, gestational age, birth weight, pregnancy history, and family medical history, are summarized in Table 1.

**TABLE 1:** Characteristic of selected cases of Idiopathic and Isolated Late‐Maturation of B Cells.

Patient	Gender	Ethnicity	Gestational Age (weeks)	Weight at birth (grams)	Pregnancy	Family History	Clinical
1	M	Caucasian	40	2870	IVF‐ET; Fetal growth arrest; Meconium‐stained fluid; Positive GBS screening	Mother: Hypothyroidism in therapy; Father's aunt: Brugada syndrome	Hypertelorism; Receding chin with carp‐like mouth attitude; Pilonidal dimple with hairs.
2	M	African	39	3060	GDM treated with diet	Brother: Single kidney	None
3	M	Caucasian	41	2990	Gestational hypothyroidism in Levothyroxine therapy	Mother: Hypothyroidism in therapy, allergic to inhalants and foods; Paternal grandmother: Multiple sclerosis	Hypospadias
4	M	Caucasian‐Asian	40	4210	Hyperglycaemia	Brother: FUP for Lymphadenomegaly	Dolichocephaly; eczema
5	F	Caucasian	40	2780	Problem‐free pregnancy	Mother: Celiac disease	None
6	M	Caucasian	38	3100	Problem‐free pregnancy	Mother: Allergic to amoxicillin	Sacral vascular lesion
7	M	Caucasian‐Hispanic	39	3200	Gestational hypothyroidism in (Levothyroxine) therapy; Prenatal US identification of right–left disproportion.	Mother: Thyroid nodule; Father: Pharyngo‐laryngeal genetic anomaly	Left pre‐ear appendage
8	M	Caucasian	39	2820	Genital herpes (acyclovir); risk of preeclampsia (aspirin); Gestational hypothyroidism (Levothyroxine)	Parents: Recurrent genital herpes	None
9	M	Hispanic	37	3800	Problem‐free pregnancy	Mother: Allergic to penicillin	None
10	F	Caucasian	40	3200	Klebsiella‐UTIs; antibiotics therapy at birth	Parents: Allergic to inhalants; Daughter of father's cousin: Polydactyly; Maternal grandmother: hyperthyroidism	None
11	M	Caucasian‐African	40	2755	Problem‐free pregnancy	Father: DMII	None
12	M	Caucasian	41	3450	Homologous ICSI; aspirin and progesterone therapies	None	None
13	M	Caucasian	39	3210	Heterologous ICSI	None	Left ear fistula
14	M	Caucasian	40	3650	Hypothyroidism in therapy; TVR pos treated with vancomycin	Grandmother: Hashimoto Thyroiditis	None
15	F	Bosnian	38	2700	Antibiotic therapy for pharyngitis during pregnancy	None	None
16	F	Caucasian	41	3150	None	Mother: Hashimoto, Mother's aunt: Diabetes autoimmune	None
17	M	Caucasian	38	3447	None	Father: Carrier of cystic fibrosis; Mother: Autoimmune hypothyroidism	None
18	M	Caucasian‐Hispanic	39	3120	GDM, suspect ICP, urgent c‐section for hypo variable CTG	None	Laterocervical lymphadenopathies (resolved at 1 month of life)

Abbreviations: CTG, Cardiotocography; FUP, Follow‐Up; GBS, Group B Streptococcus; GDM, Gestational Diabetes Mellitus; ICP, Intrahepatic cholestasis of pregnancy; IVF‐ET, In Vitro Fertilization and Embryo Transfer; N.A., Not Available; US, Ultrasound; UTIs, Urinary Tract Infections.

The cohort consisted of 18 patients, predominantly male (77.8%). In terms of ethnicity, 11 of the 18 subjects were born to Caucasian parents, while four cases were of mixed ethnicity with one Caucasian, one case was of African, one Bosnian and one Hispanic ethnicity.

Regarding gestational age (mean gestational age 39 weeks, SD ± 1.1) and birth weight (mean weight 3195 grams, SD ± 384.9), all 18 newborns were born at term with normal weights in accordance with the inclusion criteria. Most of the pregnancies (15 out of 18) were conceived naturally, with three pregnancies by In Vitro Fertilization and Embryo Transfer. Various pregnancy‐related complications were found in 12 cases, including gestational diabetes, hypothyroidism requiring treatment and other conditions such as fetal growth restriction, intrahepatic cholestasis of pregnancy, and urinary tract infections. Seven out of 18 patients had a family history of autoimmune conditions such as coeliac disease, hypothyroidism, or multiple sclerosis, while four cases had no relevant family history; none had a history of immunodeficiency disorder. Clinically, 11 out of 18 patients had no abnormalities. The remaining cases (39%) had a few minor congenital abnormalities, including facial changes, hypospadias, and laterocervical lymphadenopathy.

### 3.2 Characteristics and investigations conducted during V1 and V2

The average age at V1 was 13.5 days (SD ± 5.4). The percentage of B cells (CD19+) by flow cytometry was found to be less than or equal to 2%, with an average of 54 circulating B cells per microliter of blood (SD ± 38.8).

Among all cases, serum immunoglobulins showed normal representation of IgG at this age expected to be largely maternally derived, (mean value 840 mg/dL, SD ± 146.8), absent IgA (in all cases except one), and absent or low levels of IgM (mean value 6 mg/dL, SD ± 11.8). KREC values in the DBS_V1 have a number of copies >231 in the 78% of cases with an average copy number of 551copies/cell × 10^5^ (SD ± 670.7).

At V2, the average age was 50.9 days (SD ± 16.3). Flow cytometry revealed a mean percentage of B cells (CD19+) of 18.0% (SD ± 5.5), with an average of 1177 circulating B cells per microliter of blood (SD ± 490). At V2, serum immunoglobulins showed IgG levels within the expected maternal range, while IgA became detectable in 7/18 newborns (mean 4.7 mg/dL, SD ± 6.4) and IgM in 17/18 newborns (mean 20.8 mg/dL, SD ± 14.8). In cases with abnormal KRECs at DBS_V1, testing was repeated at V2 on whole blood; all results were within the normal range.

**FIGURE 2:**
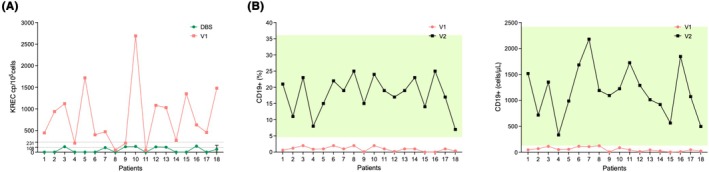
Longitudinal KREC and CD19^+^ B‐cell assessment. (A) KREC values at screening, V1, and V2; dashed lines indicate the center‐specific diagnostic cutoffs used in Tuscany and Liguria: 231 and 108 copies/10^5^ cells, respectively. For patient 18, an additional second DBS KREC measurement is shown according to the Liguria protocol described in Figure 1. (B) CD19^+^ B‐cell percentages and absolute counts at referral visit (V1) and follow‐up visit (V2). Green shaded areas indicate the age‐adjusted 0–2‐month reference ranges ([4.6%–36.1%]; [127–2.418 cells/μL]) adapted from Borriello et al.^21^ DBS, dried blood spot; KREC, kappa‐deleting recombination excision circles; V1, referral visit at the tertiary immunology center; V2, follow‐up visit.

Four cases with genetically confirmed agammaglobulinemia served as positive controls and consistently exhibited failure to normalize both KREC values and CD19+ values over the course of follow‐up. Details are described in Table 2 and Figure 2.

**TABLE 2:** Age, B cells (% and cells count), immunoglobulin levels (mg/dL) for each patient at V1 and V2.

Patient	Age V1 (days)	B‐cells (%)	B‐cells (cells/μL)	IgG (mg/dL)	IgA (mg/dL)	IgM (mg/dL)	Age V2 (days)	B‐cells (%)	B‐cells (cells/μL)	IgG (mg/dL)	IgA (mg/dL)	IgM (mg/dL)
1	14	0.6	45	875	0	7	48	21	1516	459	0	8
2	14	1.2	69	772	0	0	52	11	716	385	0	20
3	16	2	112	772	0	20	93	23	1351	269	9	37
4	12	0.9	49	924	0	18	40	8	332	477	17	18
5	18	1	56	629	0	13	37	15	986	349	13	63
6	18	2	114	660	0	0	46	22	1683	322	0	7
7	11	1	108	755	0	0	43	19	2179	319	11	34
8	11	2	123	834	0	0	85	25	1190	258	0	28
9	13	0.1	5	1037	0	0	56	15	1092	529	9	21
10	11	2	86	1189	0	7	44	24	1224	672	0	15
11	18	1	44	605	0	0	54	19	1725	306	0	9
12	11	0.2	11	856	0	0	53	17	1288	458	0	17
13	9	1	43	908	0	0	33	19	1013	612	0	8
14	9	1	24	676	0	0	39	23	919	406	6	33
15	11	0	2	993	0	0	44	14	563	375	0	7
16	6	0	9	959	0	0	33	25	1846	460	0	0
17	10	1	46	857	0	0	50	17	1072	376	0	16
18	31	0.4	23	817	50	47	66	7	497	572	25	26

All patients were contacted via telemedicine, and no new health concerns were reported over a total of 39.5 *person‐years of follow‐up* (*mean 2.3 ± 1.7 years*).

## 4 DISCUSSION

The introduction of KREC quantification into NBS programs has expanded the ability to identify newborns with early disturbances of B‐cell development.^22^ However, the clinical relevance and optimal management of newborns presenting with isolated KREC abnormalities remain incompletely defined. Our findings contribute to this discussion by highlighting the complexity of interpreting KREC results in early life and by illustrating both the potential advantages and the limitations of different follow‐up strategies.^23^, ^24^

From a biological perspective, the dissociation observed in our cohort between the rapid normalization of KREC values on repeat DBS testing and the persistence of marked peripheral B‐cell lymphopenia is biologically plausible. KREC levels reflect recent bone marrow B‐cell output rather than the size of the circulating B‐cell pool; therefore, recovery of KREC values may precede the restoration of age‐appropriate peripheral B‐cell counts. In this setting, normalization of KREC values in the presence of ongoing B‐cell lymphopenia may represent an early sign of delayed, yet ultimately transient, maturation of the B‐cell compartment, a pattern consistently reported across international KREC‐based NBS programs.^9^, ^10^, ^12^, ^25^, ^26^, ^27^ More recently, the Dutch group proposed second‐tier molecular testing to reduce false‐positive KREC referrals.^26^ However, these reports did not systematically assess peripheral B‐cell counts and antibody production during follow‐up, limiting the characterization of this transient phenotype.

These observations raise important considerations regarding current NBS algorithms. A management strategy based exclusively on repeat DBS testing may fail to identify a subset of newborns with persistent, albeit transient, B‐cell lymphopenia whose KREC values have already normalized at the time of recall. In our series, these newborns could only be retrospectively classified once peripheral B‐cell counts had returned to the normal range. Early clinical assessment and lymphocyte subset analysis allowed a more accurate characterization of the condition and informed short‐term clinical decision‐making. In our cohort, most infants were reassessed (V2) approximately 1 month (36.0 ± 17.4) after V1, a timeframe that appeared sufficient to document peripheral B‐cell recovery and the emergence of immunoglobulin production (Table 2).

Persistent B‐cell lymphopenia during early infancy may have practical implications for vaccination planning. International recommendations advise caution with live vaccines, including oral rotavirus vaccine, in infants with suspected or confirmed significant immunodeficiency. Pending confirmation of B‐cell recovery and exclusion of major antibody defects, temporary individualized deferral of rotavirus vaccination may therefore be considered in collaboration with immunologists and primary care pediatricians.^28^, ^29^

At the same time, several arguments caution against systematic follow‐up of all newborns with isolated KREC abnormalities. In many cases, B‐cell lymphopenia appears to be self‐limited and clinically silent, with spontaneous normalization of peripheral B‐cell counts and no apparent increase in infectious susceptibility. Universal referral and prolonged monitoring may therefore result in overdiagnosis and unnecessary medicalization of a condition that ultimately resolves without intervention.

Moreover, the potential psychosocial impact on families must be acknowledged.^30^ Early referral to immunology services and repeated investigations may increase parental anxiety, particularly when the probability of spontaneous resolution is high. From a public health perspective, NBS programs aim to maximize benefit while minimizing harm, and excessive follow‐up of newborns with transient abnormalities may not align with these principles. In addition, the feasibility and sustainability of widespread specialist referral must be considered, particularly in healthcare systems with limited access to pediatric immunology services.

Although no universally accepted post‐screening algorithm exists for isolated low KREC, repeat DBS testing before specialist referral is incorporated in several international NBS programs to reduce false‐positive recalls.^10^, ^12^, ^13^ In line with these approaches, and although based on a limited number of cases, our experience suggests that repeating KREC quantification on a second DBS collected within the first 2 weeks of life may help refine the initial risk stratification of newborns with isolated KREC abnormalities. Indeed, when only infants with persistently low KREC values after repeat DBS testing are considered, the proportion of newborns requiring further evaluation appears comparable between the Tuscany and Liguria cohorts (8/106 (7.5%) vs. 1/18 (5.6%) respectively; Figure 1). This approach may therefore represent a pragmatic strategy, potentially reducing unnecessary referrals while still identifying newborns with abnormalities who may benefit from early immunological assessment. At present, neither a conservative approach based solely on repeat DBS testing nor a more proactive strategy involving systematic early referral can be regarded as clearly superior. Each approach has potential benefits and drawbacks, and their relative value remains uncertain in the absence of robust long‐term outcome data.

Isolated KREC abnormalities likely represent a heterogeneous biological phenomenon, encompassing newborns with transient and clinically irrelevant delays in B‐cell maturation as well as others in whom early lymphopenia may have short‐ or long‐term immunological implications that are not yet fully understood. B lymphocytes' immunoglobulin somatic hypermutation and class switch recombination occur already during intrauterine life, with evidence of KRECs already at 12 weeks of gestational age.^31^ However, B cell representation in peripheral blood increases during the third trimester (29–40 weeks) with studies showing low percentages up to Week 27.^32^ It is therefore possible that our observation might reflect the low end of normal physiological variability. Distinguishing between these trajectories in early infancy remains challenging, and current screening tools alone are insufficient to reliably predict individual outcomes.

Only longitudinal follow‐up studies will be able to determine whether newborns with transient B‐cell lymphopenia detected through KREC‐based NBS are at increased risk of clinically meaningful consequences, such as impaired vaccine responses, increased infectious morbidity, or subtle long‐term alterations of humoral immunity. Prospective studies integrating serial immunophenotyping, assessment of antigen‐specific antibody responses following routine immunizations, and standardized clinical follow‐up– ideally within multicenter and international collaborative frameworks– will be essential to address these questions.

Until such evidence becomes available, management of newborns with isolated KREC abnormalities should remain individualized and proportionate. Our findings support repeat KREC testing on a second DBS as a useful triage step before referral, followed by targeted immunological assessment in newborns with persistently abnormal results or clinical concerns. This approach may help balance early identification of clinically relevant IEI with the need to minimize unnecessary referrals, parental anxiety, and overmedicalization.

The principal limitation of our investigation is the small sample size, which reflects the limited adoption of KREC testing in NBS for IEI in only a few Italian regions; larger, multinational cohorts will be required to validate and extend these preliminary observations.

## AUTHOR CONTRIBUTIONS

**Traverso Monica:** Methodology; writing – review and editing. **Pelosi Caterina:** Methodology; writing – review and editing. **Schena Francesca:** Methodology; writing – review and editing. **Boscia Silvia:** Methodology; writing – review and editing. **Guarnieri Valentina:** Conceptualization; data curation; writing – review and editing; writing – original draft; methodology. **Cassanello Michela:** Writing – review and editing; methodology. **Drago Enrico:** Conceptualization; data curation; writing – review and editing; methodology. **Astorino Valeria:** Methodology; writing – review and editing. **Salina Alessandro:** Methodology; writing – review and editing. **Volpi Stefano:** Supervision; project administration; writing – review and editing; funding acquisition; conceptualization. **Azzari Chiara:** Supervision; writing – review and editing; funding acquisition; project administration. **Palmeri Serena:** Methodology. **Aloi Concetta:** Methodology; writing – review and editing. **Lodi Lorenzo:** Writing – review and editing. **Ricci Silvia:** Conceptualization; writing – review and editing; writing – original draft; funding acquisition; supervision; project administration. **Maghnie Mohamad:** Writing – review and editing; funding acquisition; project administration; supervision.

## FUNDING INFORMATION

This research did not receive any specific grant from funding agencies in the public, commercial, or not‐for‐profit sectors.

## CONFLICT OF INTEREST STATEMENT

The authors have no relevant financial or non‐financial interests to disclose.

## Data Availability

The data that support the findings of this study are available from the corresponding author upon reasonable request.
